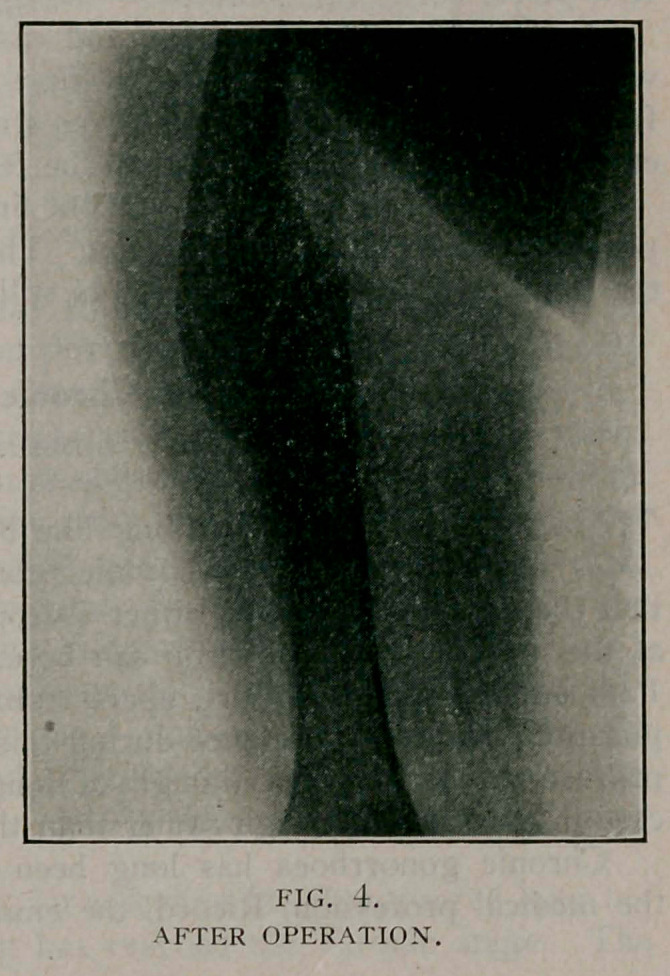# Vicious Union of the Shaft of the Femur1Read before the Buffalo Academy of Medicine, May 7, 1911.

**Published:** 1911-12

**Authors:** Edwin L. Bebee

**Affiliations:** Buffalo, N. Y.; Instructor in Surgery, University of Buffalo


### Vicious Union of the Shaft of the Femur[1]

By EDWIN L. BEBEE,, M.D.
Buffalo, N. Y.
Instructor in Surgery, University of Buffalo

BY vicious union we mean union with such a degree of rotation, bowing and shortening as to result in permanent limp or deformity. The affected thigh is short and crooked; there is tilting of the pelvis and lowering of the shoulder on the affected side. This is due to the small size of the shaft of the femur in the middle third where fracture usually occurs: to the oblique direction of the surface of fracture, which is smooth so that there is rarely or never an interlocking of the fragments. But there is great overriding; due to spasmodic contraction of the very strong muscles of the thigh, and the change in the shape of the tubular fascia lata which becomes shortened by its distention with blood and serum. If this overriding is not corrected, the resulting callus, though large, works at a disadvantage, and bowing results, when the patient begins to bear weight on the leg, even after a long period of immobilization.

How much of this deformity is compatible with good practice is still under discussion. In 1890 a committee of the American Surgical Association led by Stephen Smith decided that it was allowable to have one inch of shortening, and permanent lameness if not due to more than that amount of shortening. But today there is a school of surgeons who hold that anatomically perfect results should be obtained by means of open operation and mechanic fixation. These are the radicals, with Lane of London in the forefront as a leader. They advise operation in all simple fractures. Not all are willing to go so far.

Jonas of Omaha says: "Those of us who had an opportunity of examining Lane's numerous skiagrams of simple fractures on which operation had recently been done, could not help but be impressed with the fact that some of his cases would have done quite as well if the fractures had been reduced by manipulation, and the use of the same external splints that he employed to reinforce the clips and screws which he used through an incision."

Dr. Robert F. Weir of New York after long experience in the treatment of fractures by the conservative method, considers that the results have been satisfactory and more than counterbalance the risk of an open operation.

Ashurst reports in the *Annals of Surgery,* that of 22 cases treated conservatively at the Episcopal Hospital in Philadelphia,

---

1, Read before the Buffalo Academy of Medicine, May 7, 1911.

and observed later, 14 had perfect results; only 8 had a limp; 5 had a shortening of 2 c.m.; 8 had a shortening of 1 c.m.

Dr. Carlton P. Flint of New York, writes that from September, 1906 to October 1907, there came under his observation at Roosevelt Hospital, New York, 834 cases of fracture of the femur; of these 53 were operated, 27 after delay for corrective purposes. So that while we may not care to operate on every recent case of fracture, still there is a class of cases, which, after conservative treatment has been tried, call for operation, on account of persisting deformity and disability.

In the correction of such a deformity wide incision is necessary, exposure of the point of mal-union, separation of the fragments by chiseling through the callus, freshening the ends of the fragments, and fastening them by some mechanic device; and then supporting the parts by suitable external splints.

Of the various means of fixation, as wire, screws, nails, plates and clamps, they all have the disadvantage of acting as foreign bodies which work against union, often prevent it, and have to be removed. When introduced into bone they cause a rarifying osteitis, so that they become loosened and fail to hold in from one to two weeks. Wire has the disadvantage of giving retention only in one plane. Silver wire breaks easily, and has been replaced by aluminum bronze.

The rigid plate of Lane has the disadvantage, when acted upon through the leverage of the extremity by an accidental movement, of easily wrenching out the screws. This is obviated in the flexible silver plate of Sick. This comes long and can be cut to a suitable length. Intra-medullary splints displace the bone marrow, which has an important function in callus formation, and when of ivory or metal act as foreign bodies, which may have to be removed. So we have usually preferred absorbable ligatures of chromic gut or kangaroo tendon.

Freeman reports that out of 18 cases operated on, delayed union resulted in 9 cases with non-union in two of these. Wire was employed in 6, a clamp in one. Six of these, however, were in compound fractures, and two had no foreign material introduced.

**Case Report**

GIRL, AGE 6. REFERRED BY DR. PRESCOT LE BRETON.

On April 13, 1910, she suffered a fracture of the shaft of the right femur from being stepped on by a horse. She was treated by some form of splint without extension for two weeks. After nine weeks she was allowed to bear weight on the leg. This

FIG. 1.

BEFORE OPERATION.

FIG. 3.

BEFORE OPERATION.

FIG. 2.

AFTER OPERATION.

FIG. 4.

AFTER OPERATION.

was followed by bowing. Her parents sought relief from the resulting limp and deformity.

June 30. On examination she walked with a marked limp, carrying the right shoulder and hip low. There was a forward and outward bowing in the right thigh. There was an extensive mass of callus at the point of union. The right leg was 1¼ inches shorter than the left.

July 6. Operation. An incision four inches long was made on the outer aspect of the right thigh opposite the most prominent part of the deformity. This was carried down to the bone, exposing the overlapping fragments held together by extensive callus. The fragments were separated by chiseling through the callus. The bone ends were freshened with chisel and bone forceps, making the surfaces to be opposed oblique in direction. A hole was drilled through the fragments at right angles to the freshened surfaces, for the introduction of a ligature of chromic gut. The muscles were brought together with interrupted sutures of plain cat-gut, and the skin with the same material. A plaster of Paris spica reaching to the knee, was applied. During the insertion of the bone ligature, manual traction was made to overcome the shortening. This was replaced by a weight of ten pounds attached by means of strips of adhesive to the leg with foot piece, cord, and pulley.

August 10. The extension and cast were removed and the wound dressed. Healing was by first intention. Union in the fragments was firm. The thigh was straight with ¼ inch shortening. Considerable callus was to be felt.

October 27. She walked without limp and presented the appearance shown in the photograph. There was some atrophy of the thigh and calf. The callus was still to be felt.

## Figures and Tables

**Fig. 1. f1:**
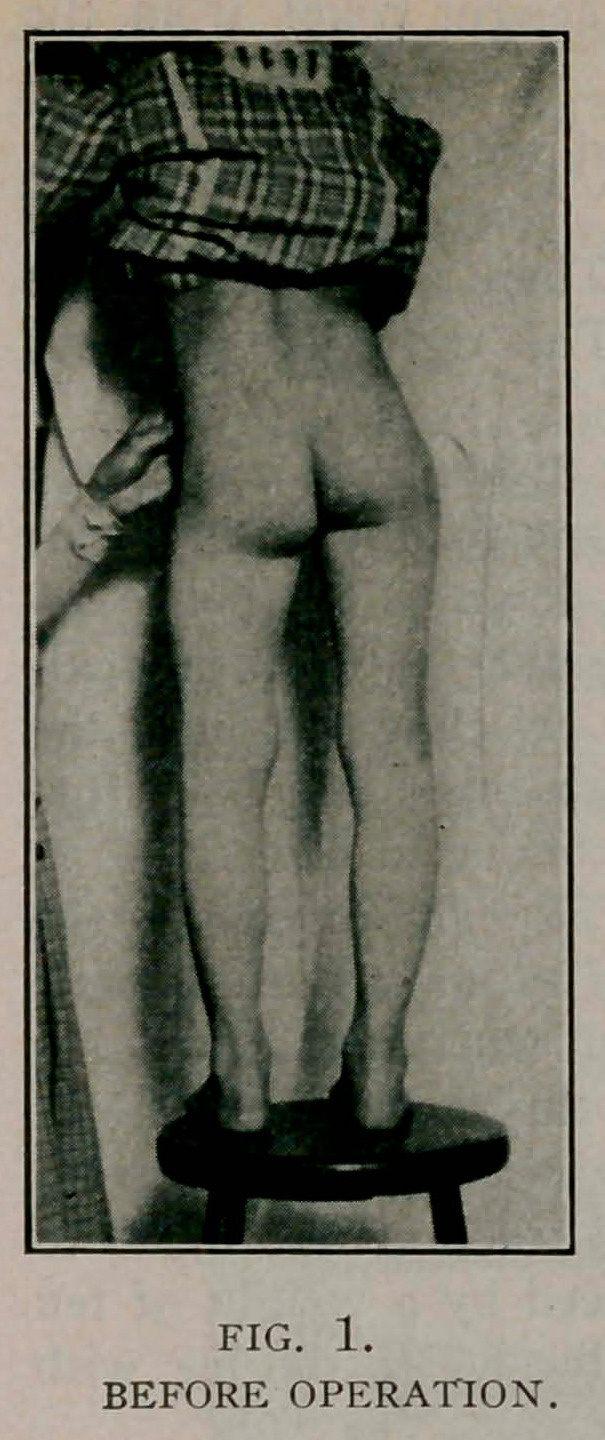


**Fig. 2. f2:**
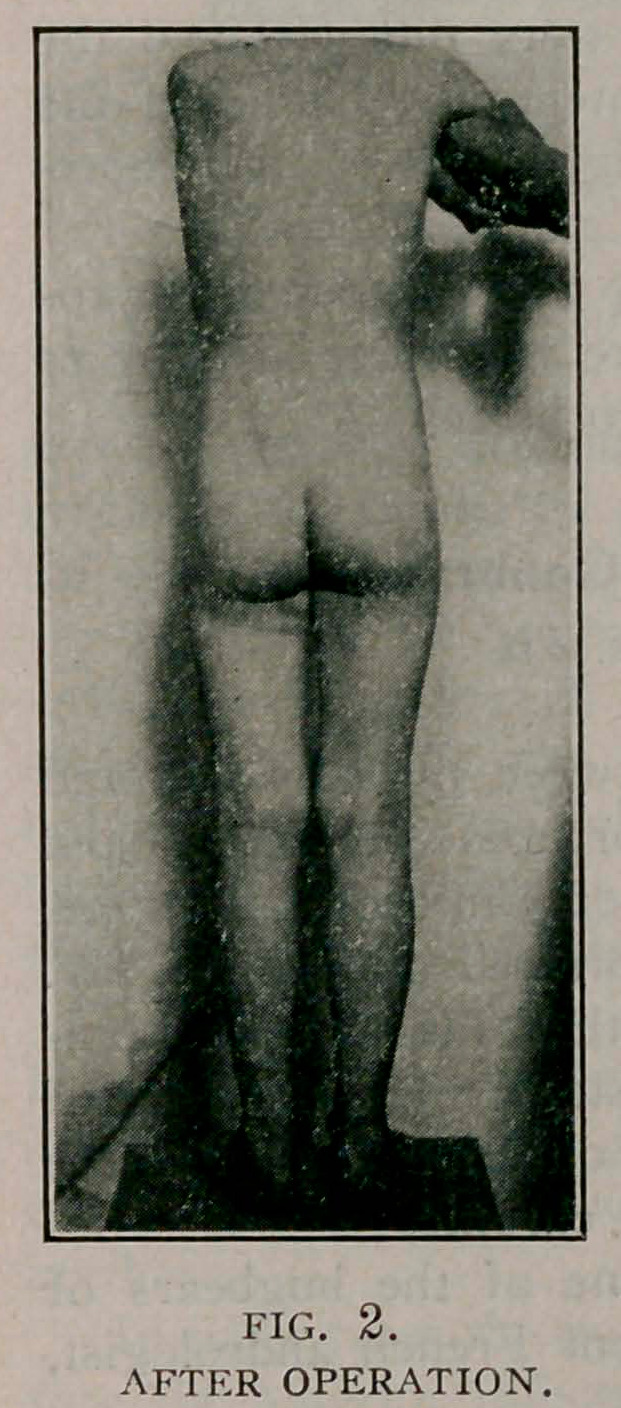


**Fig. 3. f3:**
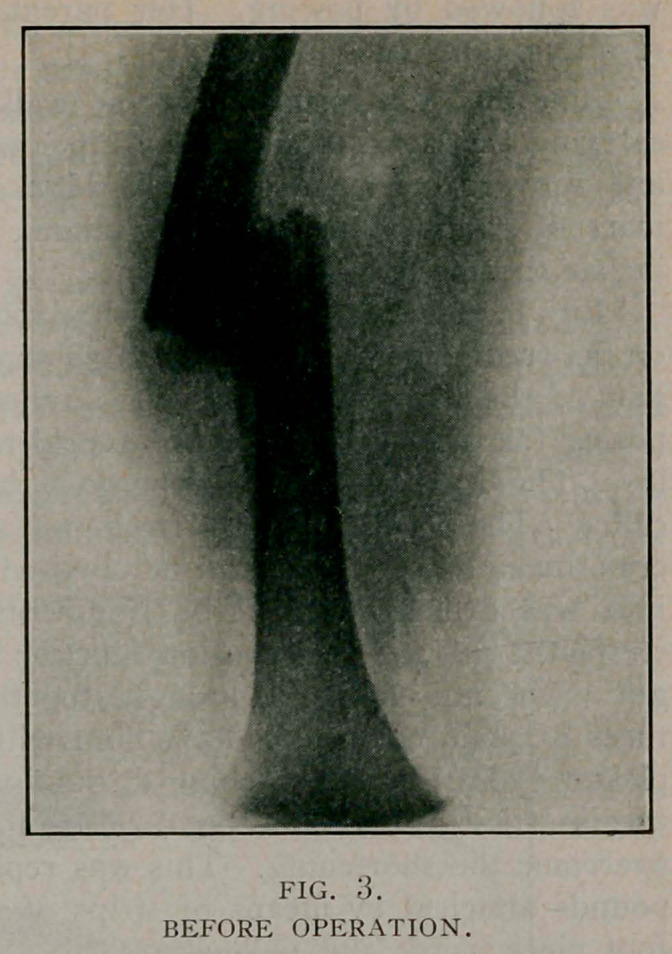


**Fig. 4. f4:**